# Antidiabetic Effect of *Monolluma quadrangula* Is Mediated *via* Modulation of Glucose Metabolizing Enzymes, Antioxidant Defenses, and Adiponectin in Type 2 Diabetic Rats

**DOI:** 10.1155/2019/6290143

**Published:** 2019-02-19

**Authors:** May N. Bin-Jumah

**Affiliations:** Department of Biology, College of Science, Princess Nourah bint Abdulrahman University, Riyadh, Saudi Arabia

## Abstract

*Monolluma quadrangula* is a succulent bush traditionally used to treat diabetes and peptic ulcer. The present study aimed to investigate the effect of *M. quadrangula* hydroethanolic extract on glucose tolerance, insulin sensitivity, glucose metabolizing enzymes, lipid profile, and adiponectin expression in type 2 diabetic rats. In addition, the study evaluated the antioxidant and anti-inflammatory activities of the *M. quadrangula* extract. Type 2 diabetes was induced by feeding rats a high-fat diet (HFD) for 8 weeks followed by 30 mg/kg streptozotocin (STZ). Diabetic rats received 300 or 600 mg/kg *M. quadrangula* extract for 4 weeks. HFD/STZ diabetic rats showed impaired glucose tolerance, reduced insulin secretion, and insulin resistance. HFD and STZ induced a significant increase in serum cholesterol, triglycerides and proinflammatory cytokines, and liver lipid peroxidation. Treatment with *M. quadrangula* extract ameliorated these metabolic disturbances and increased liver glycogen, hexokinase activity, and antioxidants. *M. quadrangula* declined the activity of liver glucose-6-phosphatase and fructose-1,6-biphosphatase. In addition, *M. quadrangula* extract increased serum adiponectin levels and hepatic adiponectin expression in HFD/STZ diabetic rats. In conclusion, *M. quadrangula* exerts antidiabetic effect mediated *via* ameliorating glucose tolerance, insulin sensitivity, glucose metabolizing enzymes, and antioxidant defenses. Increased adiponectin levels and expression seems to mediate, at least in part, the antidiabetic effect of *M. quadrangula*.

## 1. Introduction

Diabetes mellitus (DM) is a metabolic syndrome characterized by hyperglycemia that occurs as a result of deficient insulin secretion and/or action. Uncontrolled hyperglycemia can lead to micro- and macrovascular complications, nephropathy, cardiomyopathy, and retinopathy. Type 2 DM is the common form of DM and represents a public health concern worldwide [1]. Oxidative stress, driven by hyperglycemia, plays a central role in the pathogenesis of DM and its complications [2–6]. Along with hyperglycemia, hyperlipidemia can increase the production of reactive oxygen species (ROS), leading to oxidative stress and inflammation [7, 8].

Adiponectin, a 30 kDa multimeric protein, is secreted mainly by adipose tissue; however, it is expressed in the liver, myocytes, placenta, epithelial cells, and osteoblasts [9–12]. Several studies have demonstrated the role of adiponectin as a regulator of glucose and lipid metabolism, insulin sensitivity, and cardiovascular homeostasis. In humans, lowered levels of adiponectin are associated with the development of obesity and type 2 DM as well as cardiovascular disease [13]. Through its action in the hypothalamus, adiponectin plays an important role in energy homeostasis [14]. In the skeletal muscle of high-fat-/sucrose diet-fed mice, adiponectin increased the fatty acid oxidation and glucose uptake [15]. Adiponectin protected against cerebral ischemia-reperfusion [16] and atherosclerotic plaque formation [17] and improved revascularization of ischemic limbs [18]. In high-fat diet- (HFD-) fed mice, adiponectin overexpression improved the metabolic flexibility and prevented the lipotoxic effects of lipid accumulation [19]. In primary rat hepatocytes, adiponectin decreased the glucose output as reported by Berg et al. [20]. These studies show that increasing adiponectin is an attractive target for the treatment of type 2 DM.

The present study aimed to investigate the antihyperglycemic and insulin sensitizing effects of *Monolluma quadrangula* (Forssk.) extract, focusing on its role in modulating glucose metabolizing enzymes, oxidative stress, and adiponectin expression. *M. quadrangula* is a succulent bush with a yellow flower and irregularly branched and a compressed stem [21]. It is known as *Caralluma quadrangula* and has been used in folk medicine for the treatment of DM and peptic ulcer. *M. quadrangula* showed an antioxidant effect in ethanol-induced peptic ulcer [22] and high-cholesterol diet- (HCD-) fed rats [23]. Recently, we reported that *M. quadrangula* ameliorated serum lipids, hepatic and cardiac oxidative stress, and the expression of fatty acid synthase (FAS) and low density lipoprotein- (LDL-) receptor in HCD-fed rats [23]. Therefore, *M. quadrangula* could be a promising candidate for the treatment of diabetes. The antihyperglycemic effect of methanol, chloroform, and n-butanol extracts of *M. quadrangula* has been recently tested by Abdel-Sattar et al. [24] which showed a decreased fasting blood glucose, insulin, and glucose-phosphatase in streptozotocin- (STZ-) induced diabetic rats. However, the effect of *M. quadrangula* on glucose tolerance, insulin sensitivity, lipid profile, oxidative stress, and adiponectin expression in type 2 DM has not been investigated.

## 2. Materials and Methods

### 2.1. Collection of *M. quadrangula* and Extract Preparation

The collection of *M. quadrangula* samples and preparation of hydroethanolic extract were conducted as we recently described [23]. Briefly, *M. quadrangula* collected from Abha-Al-Taif road (Saudi Arabia) were air-dried, grounded into fine powder in an electric grinder, and soaked for 24 h in water/ethanol (1 : 1 vol/vol). After filtration of the mixture and evaporation of the solvent in a rotary evaporator, the dried residue was collected and used for animal treatments.

### 2.2. Experimental Induction of HFD/STZ Diabetes and Treatment with *M. quadrangula* Extract

Male Wistar rats were fed a HFD *ad libitum* for 8 weeks and then received a single intraperitoneal (ip) injection of STZ (30 mg/kg) dissolved in freshly prepared cold citrate buffer (pH 4.5). Seventy-two h after STZ injection, blood glucose was measured and rats having fasting blood glucose of more than 200 mg/dL were considered diabetic. A corresponding group of rats fed with a normal diet and received a single ip injection of citrate buffer served as a control group.

All animals included in this study were obtained from the animal house of King Saud University (Saudi Arabia), and all procedures were approved by the ethical committee at Princess Nourah bint Abdulrahman University (Riyadh, Saudi Arabia). The animals were housed under standard laboratory conditions as we previously reported [23].

The total of six normal control rats was used as group I (control) and received a daily dose of distilled water via oral gavage for 4 weeks. The diabetic rats were allocated randomly into 3 groups, each group has six, as follows: group II (diabetic) included diabetic rats which received distilled water orally and daily for 4 weeks, group III and group IV included diabetic rats which received daily doses of 300 and 600 mg/kg *M. quadrangula* extract dissolved in distilled water via oral gavage for 4 weeks [23].

At the end of the experiment, all groups were fasted overnight and sacrificed under anesthesia. Blood samples were collected to separate the serum and the rats were dissected to collect the liver. Samples from the liver were homogenized in a cold 0.1 M phosphate buffer (pH 7.4), centrifuged at 8000 rpm, and used for biochemical assays. The other samples were kept frozen at -80°C for RNA isolation.

### 2.3. Glucose Tolerance Test

Oral glucose tolerance test (OGTT) was performed on the day before the sacrifice. Overnight-fasted rats received 3 g/kg glucose solution orally, and blood samples were collected from the tail vein at 30, 60, 90 and 120 min [5]. Glucose levels were assayed in the serum prepared from the collected blood samples using kits supplied by SPINREACT (Spain) [25].

### 2.4. Determination of Serum Lipids and Cardiovascular Risk Indices

Total serum cholesterol [26], HDL-cholesterol [27], and triglycerides [28] were assayed using Accurex kits (Mumbai, India). LDL- and vLDL-cholesterol were then calculated as follows: vLDL − cholesterol = triglycerides/5 and LDL − cholesterol = total cholesterol–(HDL − cholesterol + vLDL − cholesterol).

### 2.5. Assay of Serum Insulin, Proinflammatory Cytokines, and Adiponectin

Serum insulin, interleukin-6 (IL-6), tumor necrosis factor alpha (TNF-*α*), and adiponectin were assayed using ELISA kits (Merck Millipore), USA.

### 2.6. Assay of Liver Glycogen, Hexokinase, Glucose-6-Phosphatase, and Fructose-1,6-Biphosphatase

Liver glycogen [29], hexokinase [30], glucose-6-phosphatase [31], and fructose-1,6-biphosphatse [32] were assayed in the liver homogenate of control and diabetic rats.

### 2.7. Calculation of Homeostasis Model of Insulin Resistance (HOMA-IR)

HOMA-IR was calculated using insulin and glucose measurements as follows [33]:

HOMA − IR = fasting insulin (*μ*U/ml) × fasting glucose (mmol/L)/22.5.

### 2.8. Assay of Lipid Peroxidation, Glutathione, Superoxide Dismutase, and Catalase

Lipid peroxidation, GSH, superoxide dismutase (SOD), and catalase (CAT) were assayed in the liver homogenate using OxiSelect kits (USA).

### 2.9. Assay of Adiponectin Gene Expression

To analyze the gene expression levels of adiponectin in the liver, we used qPCR as previously described [23, 34]. In brief, RNA was isolated using a Bioline RNA Mini kit (USA). The extracted RNA was quantified on NanoDrop 8000 (Thermo Scientific, USA) and samples with 1.8-2.0 260/280 absorbance ratio were used for reverse transcription into cDNA. The prepared cDNA was amplified using SYBR Green Master Mix (Invitrogen, USA) and primer pairs supplied by metabion international AG (Germany) (Table 1). The amplification data were analyzed using the 2^-*Δ*ΔCt^ method [35].

### 2.10. Statistical Analysis

The results were analyzed using GraphPad Prism (GraphPad Software, CA, USA). The results were presented as mean ± standard error (SEM), and one-way ANOVA test followed by Tukey's test was used for statistical comparisons. A *P* value less than 0.05 was considered significant.

## 3. Results

### 3.1. *M. quadrangula* Attenuates Hyperglycemia in HFD/STZ Diabetic Rats

**Figure 1:**
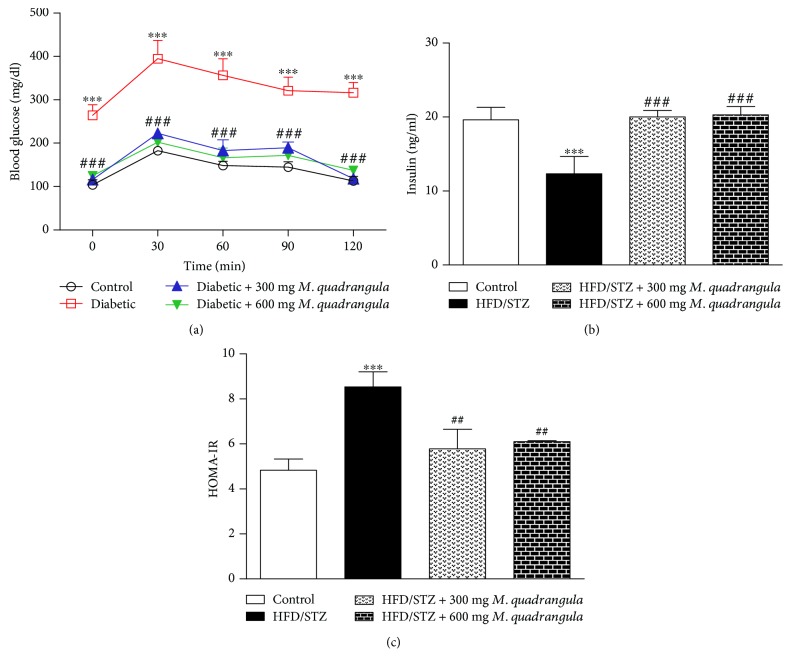
Effect of *M. quadrangula* extract on (a) glucose tolerance, (b) serum insulin, and (c) HOMA-IR value of HFD/STZ type 2 diabetic rats. Data are mean ± SEM. The number of animals in each group is six. ^∗∗∗^*P* < 0.001 compared to control. ^##^*P* < 0.01 and ^###^*P* < 0.001 compared to HFD/STZ.

The results of glucose tolerance of HFD/STZ diabetic rats showed a significant increase in blood glucose levels at all points of the OGTT when compared with that of the control rats as represented in Figure 1(a). Oral supplementation of 300 and 600 mg/kg body weight hydroethanolic extract of *M. quadrangula* for four weeks improved the glucose tolerance as shown in the OGTT results (Figure 1(a)).

### 3.2. *M. quadrangula* Prevents Insulin Resistance in HFD/STZ Diabetic Rats

Figure 1(b) showed a significant decrease in serum insulin levels of HFD/STZ diabetic rats (*P* < 0.001) as compared to the control group. On the other hand, diabetic rats which received 300 and 600 mg/kg body weight hydroethanolic extract of *M. quadrangula* for four weeks showed improved serum insulin levels (*P* < 0.001) as compared to the diabetic control animals (Figure 1(b)).

Glucose and insulin measurements were used to calculate HOMA-IR to show the effect of *M. quadrangula* extract on insulin sensitivity as represented in Figure 1(c). Untreated diabetic rats showed a significant degree of insulin resistance (*P* < 0.001) as compared to nondiabetic rats. *M. quadrangula* extract at both 300 and 600 mg/kg doses significantly improved insulin sensitivity (*P* < 0.01) as shown by decreased HOMA-IR values (Figure 1(c)).

### 3.3. *M. quadrangula* Ameliorates Liver Glycogen, Hexokinase, Glucose-6-Phosphatase, and Fructose-1,6-Biphosphatase in HFD/STZ Diabetic Rats

**Figure 2:**
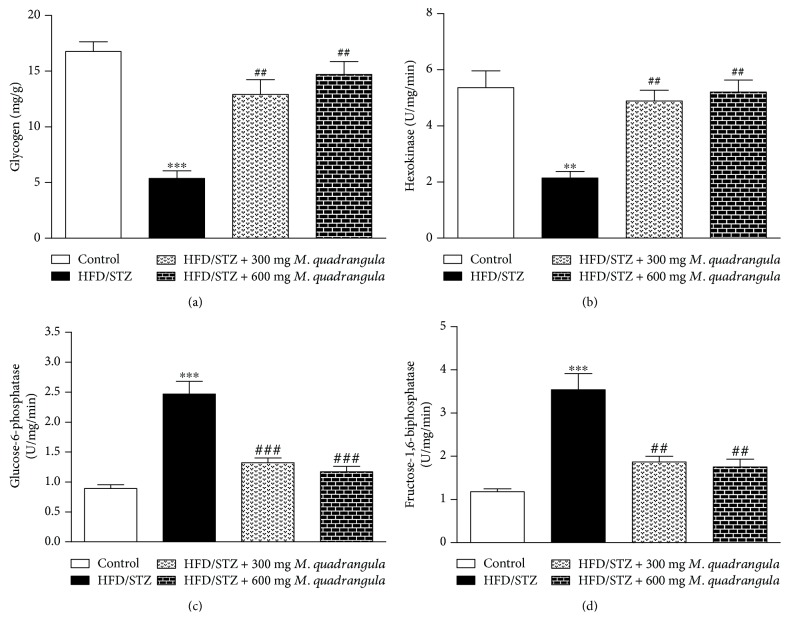
Effect of *M. quadrangula* extract on (a) liver glycogen, (b) hexokinase, (c) glucose-6-phosphatase, and (d) fructose-1,6-biphosphatase of HFD/STZ type 2 diabetic rats. Data are mean ± SEM. The number of animals in each group is six. ^∗∗^*P* < 001 and ^∗∗∗^*P* < 0.001 compared to control. ^##^*P* < 0.01 and ^###^*P* < 0.001 compared to HFD/STZ.

The data showing the effect of *M. quadrangula* extract on liver glycogen, hexokinase, glucose-6-phosphatase, and fructose-1,6-biphosphatase in the diabetic rats are represented in Figures 2(a)-2(d). HFD/STZ diabetic rats showed a significant decrease in liver glycogen content (*P* < 0.001) and in the activity of hexokinase (*P* < 0.01) as compared to the control rats as shown in Figures 2(a) and 2(b), respectively. The treatment of the diabetic rats with both 300 and 600 mg/kg *M. quadrangula* extract significantly improved (*P* < 0.01) hepatic glycogen content and hexokinase activity.

In contrast, HFD/STZ rats showed a significant increase in the activity of hepatic glucose-6-phosphatase and fructose-1,6-biphosphatase (Figure 2(c)) as compared to the control group (*P* < 0.001). The treatment of the diabetic rats with both doses of *M. quadrangula* extract significantly improved the activity of hepatic glucose-6-phosphatase (*P* < 0.001) and fructose-1,6-biphosphatase (*P* < 0.01).

### 3.4. *M. quadrangula* Ameliorates Levels of Serum Lipids in HFD/STZ Diabetic Rats

**Figure 3:**
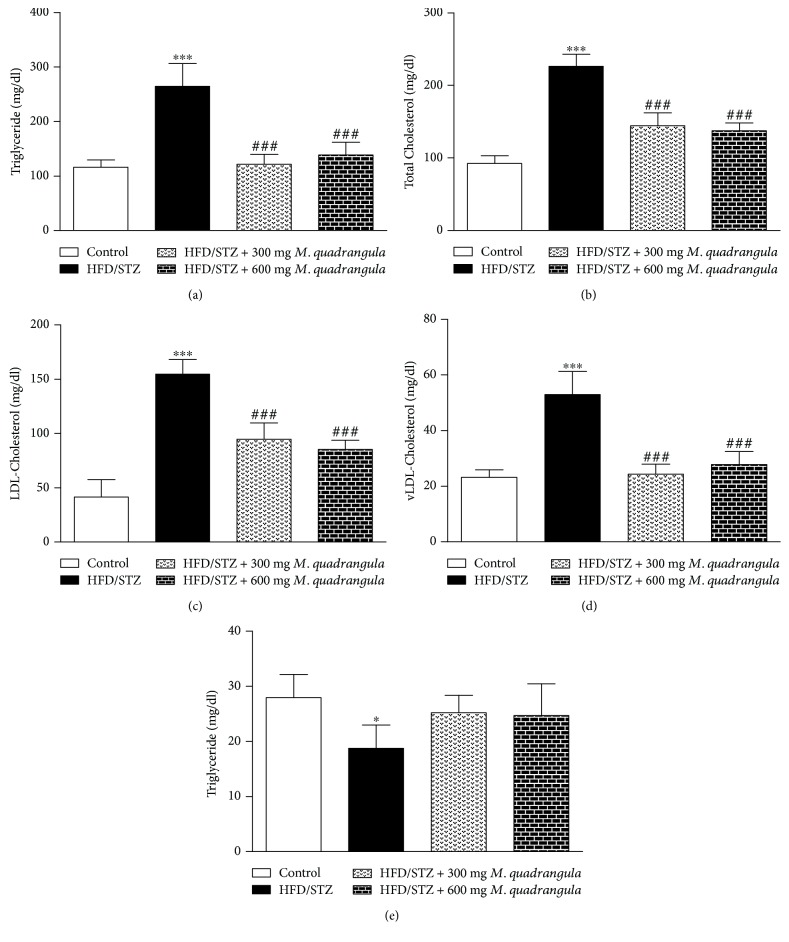
Effect of *M. quadrangula* extract on (a) serum triglycerides, (b) total cholesterol, (c) LDL-cholesterol, (d) vLDL-cholesterol, and (e) HDL-cholesterol of HFD/STZ type 2 diabetic rats. Data are mean ± SEM. The number of animals in each group is six. ^∗^*P* < 0.05 and ^∗∗∗^*P* < 0.001 compared to control. ^###^*P* < 0.001 compared to HFD/STZ.

HFD/STZ diabetic rats showed a significant (*P* < 0.001) elevation of serum triglycerides (Figure 3(a)), total cholesterol (Figure 3(b)), and LDL- (Figure 3(c)) and vLDL-cholesterol (Figure 3(d)) as compared to the control rats. The diabetic rats treated with 300 and 600 mg/kg *M. quadrangula* extract showed a significant improvement of serum triglycerides and cholesterol and its fractions (*P* < 0.001). On the other hand, HFD/STZ diabetic rats showed a significant (*P* < 0.05) decrease in serum levels of HDL and treatment with 300 and 600 mg/kg *M. quadrangula* extract did not induce significant changes as represented in Figure 3(e).

### 3.5. *M. quadrangula* Suppresses Lipid Peroxidation and Improves Antioxidants in the Liver of HFD/STZ Diabetic Rats

**Figure 4:**
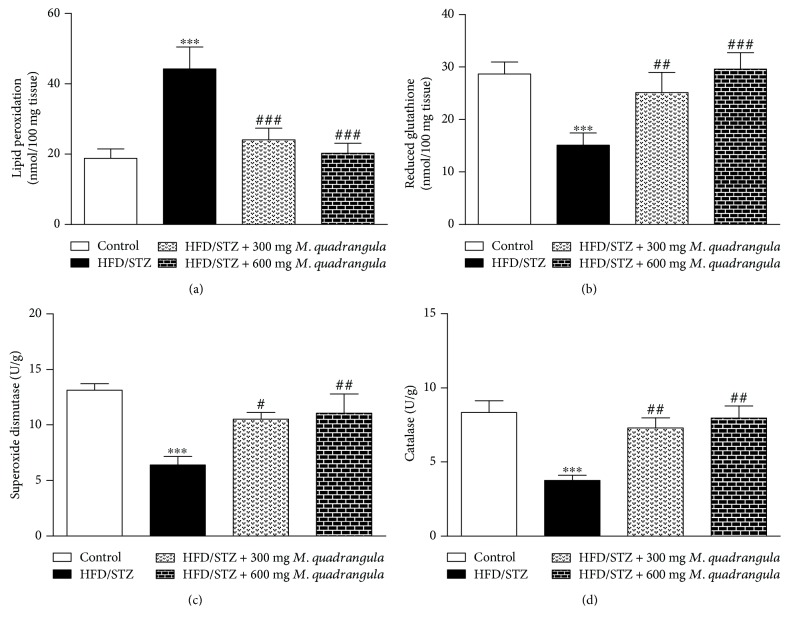
Effect of *M. quadrangula* extract on (a) liver lipid peroxidation, (b) glutathione, (c) superoxide dismutase, and (d) catalase of HFD/STZ type 2 diabetic rats. Data are mean ± SEM. The number of animals in each group is six. ^∗∗∗^*P* < 0.001 compared to control. ^#^*P* < 0.05, ^##^*P* < 0.01, and ^###^*P* < 0.001 compared to HFD/STZ.

The data presented in Figures 4(a)-4(d) showed the effect of *M. quadrangula* extract on lipid peroxidation and the antioxidants, GSH, SOD, and CAT in the liver of HFD/STZ diabetic rats. The lipid peroxidation product, malondialdehyde, showed a significant elevation in the liver of HFD/STZ diabetic rats as compared to control rats (*P* < 0.001). HFD/STZ diabetic rats treated with the two doses of *M. quadrangula* extract significantly increased the lowered liver GSH content and SOD and CAT activities.

### 3.6. *M. quadrangula* Suppresses Inflammation in HFD/STZ Diabetic Rats

**Figure 5:**
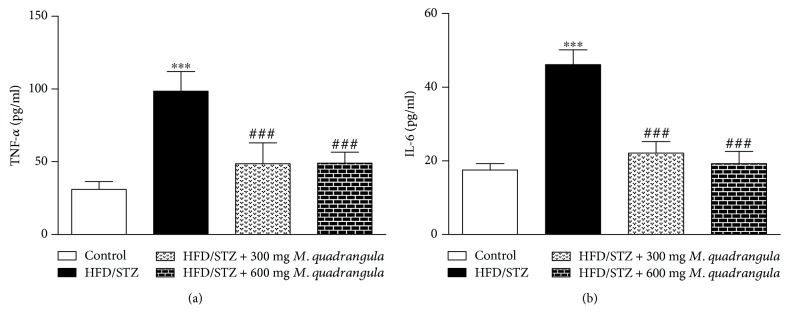
Effect of *M. quadrangula* extract on (a) serum TNF-*α* and (b) IL-6 of HFD/STZ type 2 diabetic rats. Data are mean ± SEM. The number of animals in each group is six. ^∗∗∗^*P* < 0.001 compared to control. ^###^*P* < 0.001 compared to HFD/STZ.

As presented in Figure 5, the levels of the proinflammatory cytokines, TNF-*α* and IL-6, were significantly elevated in the serum of HFD/STZ diabetic rats (*P* < 0.001) as compared to the control rats. The treatment of the HFD/STZ diabetic rats with 300 and 600 mg/kg *M. quadrangula* extract ameliorated the levels of TNF-α and IL-6 as compared to the nontreated diabetic group (*P* < 0.001).

### 3.7. *M. quadrangula* Upregulates Adiponectin in HFD/STZ Diabetic Rats

**Figure 6:**
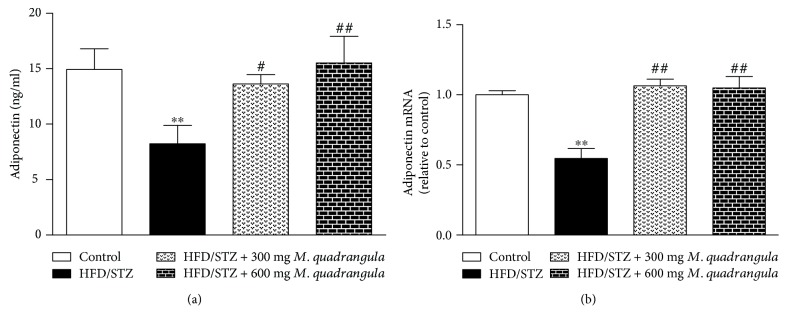
Effect of *M. quadrangula* extract on (a) serum adiponectin and (b) liver adiponectin mRNA of HFD/STZ type 2 diabetic rats. Data are mean ± SEM. The number of animals in each group is six. ^∗∗^*P* < 0.01 compared to control. ^#^*P* < 0.05 and ^##^*P* < 0.01 compared to HFD/STZ.

The data showing the effect of *M. quadrangula* extract on serum adiponectin and hepatic adiponectin gene expression are presented in Figure 6. HFD/STZ diabetic rats showed a significant decrease in serum adiponectin levels (*P* < 0.01) as compared to the control rats (Figure 6(a)). The diabetic rats which received 300 mg/kg *M. quadrangula* extract showed a significant increase (*P* < 0.05) in serum adiponectin. The 600 mg/kg *M. quadrangula* extract induced a significant increase (*P* < 0.01) in serum adiponectin levels.

Adiponectin gene expression significantly decreased (*P* < 0.01) in the liver of HFD/STZ diabetic rats as compared to the control rats as presented in Figure 6(b). The treatment with both doses of *M. quadrangula* extract increased the gene expression of adiponectin in the liver of the diabetic rats (*P* < 0.01).

## 4. Discussion

*M. quadrangula* has been used in tradition as a medicine to diabetes and peptic ulcer. However, its antidiabetic mechanisms are not known. Recently, we reported the antihypercholesterolemic effect of *M. quadrangula* in HCD-fed rats [23]. *M. quadrangula* ameliorated serum lipids, hepatic and cardiac oxidative stress, and the expression of FAS and LDL-receptor in HCD-fed rats. Given its potent lipid-lowering effect, *M. quadrangula* could be a promising candidate for the treatment of diabetes.

HFD/STZ diabetic rats in the present study showed an impaired glucose tolerance and insulin sensitivity as shown by the increased blood glucose, decreased insulin, and increased HOMA-IR value. The combination of HFD and STZ has been previously reported to induce type 2 DM characterized by an impaired glucose tolerance and insulin resistance [36–38]. Therefore, the HFD/STZ model reflects the metabolic characteristics of type 2 DM [39]. Hyperglycemia in HFD/STZ diabetic rats resulted from STZ-induced destruction of the pancreatic *β*-cells, diminished insulin secretion and sensitivity, reduced peripheral glucose uptake, and increased hepatic glucose production [40, 41]. The treatment of the HFD/STZ diabetic animals with *M. quadrangula* extract for 4 weeks improved blood glucose, insulin secretion, and insulin sensitivity, demonstrating a potent antihyperglycemic effect.

In addition to increased insulin sensitivity, we assumed that an improved peripheral glucose uptake and a decreased hepatic glucose production participate in the antihyperglycemic effect of *M. quadrangula* extract in HFD/STZ diabetic rats. Our results showed an improved hepatic glycogen content in HFD/STZ diabetic rats following the 4-week treatment with *M. quadrangula* extract. Liver glycogen is a valuable marker to evaluate the hypoglycemic effect of drugs or plant extracts [42]. In addition, the treatments with both doses of *M. quadrangula* extract increased the activity of hexokinase and decreased glucose-6-phosohatase and fructose-1,6-biphosphatase in the liver of the diabetic rats. In diabetes, the rate of glycogenolysis and gluconeogenesis increases, leading to an increased hepatic glucose output [43]. Previous studies demonstrated a decrease in hexokinase and increased activity of glucose-6-phosphatase resulting in decreased liver glycogen accompanied with hyperglycemia [39, 42]. Declined insulin secretion is another factor leading to a decreased liver glycogen because insulin activates the glycogenolytic and gluconeogenic pathways [44, 45].

The diabetic rats in the present study showed a significant increase in triglycerides, and total-, LDL- and vLDL-cholesterol in addition to a decreased HDL-cholesterol. This altered lipid profile can lead to the development of cardiovascular disease and is known as the atherogenic lipid profile [46]. In accordance with our findings, several studies have shown altered serum lipids in diabetic and insulin-resistant rats [39, 47, 48]. The atherogenic lipid profile can induce the accumulation of lipids in the liver and subsequent hepatocyte damage [49]. Recently, we have reported that rats which received a HCD for 8 weeks exhibited hepatocyte damage [23]. HFD/STZ diabetic rats which received 300 and 600 mg/kg *M. quadrangula* extract for 4 weeks showed an improvement in their lipid profile marked by a decreased serum triglycerides, and total-, LDL- and vLDL-cholesterol. There are no previous reports showing the hypolipidemic effect of *M. quadrangula* extract in diabetic rats; however, we recently reported that *M. quadrangula* decreased serum lipids in rats which received HCD for 8 weeks [23]. This improvement in serum lipids could be the result of increased serum secretion and sensitivity. We demonstrated that the modulatory effect of *M. quadrangula* on hepatic FAS and LDLR expressions mediates its hypolipidemic effect [23].

Oxidative stress driven by hyperglycemia can impair insulin signaling and induce insulin resistance. In our study, HFD/STZ-induced rats showed oxidative stress as increased liver lipid peroxidation and decreased antioxidants, GSH, SOD, and CAT. In diabetes, ROS can react and induce the peroxidation of the cell membrane polyunsaturated fatty acids, leading to cell damage. The resultant oxidative stress is an important factor in the development of diabetic complications such as nephropathy and retinopathy. Hyperglycemia can also lead to declined cellular antioxidants. In agreement with our data, Mahmoud et al. [36] reported an increased lipid peroxidation and decreased antioxidants in the liver of HFD/STZ diabetic rats. HFD/STZ diabetic rats treated with 300 and 600 mg/kg *M. quadrangula* extract for 4 weeks showed a decreased lipid peroxidation and an increased GSH, SOD and CAT. These findings agreed to the study of Ibrahim et al. [22] which showed that *M. quadrangula* extract exerts antioxidant effect in ethanol-induced gastric ulcer in rats. In this study, pretreatment with *M. quadrangula* prevented lipid peroxidation and ameliorated the gastric SOD and CAT [22]. Recently, we reported that the treatment with *M. quadrangula* extract for 8 weeks prevented oxidative stress and improved antioxidants in the liver of HCD-fed rats [23]. The antioxidant effect of *M. quadrangula* might be attributed to its rich content of glycosides and phenolics [50, 51]. In addition, the potent lipid-lowering effect of *M. quadrangula* might have a role in suppressing oxidative stress in the liver of the diabetic rats. This hypothesis is supported by studies showed that altered lipidemic status is a risk factor for oxidative stress and cell injury [7, 8].

The suppressive effect of *M. quadrangula* on oxidative stress has been associated with anti-inflammatory effect as shown by the decreased serum TNF-*α* and IL-6. HFD/STZ diabetic rats had increased serum TNF-*α* and IL-6 levels as previously reported [36]. These cytokines exert a negative impact on insulin signaling and sensitivity. TNF-*α* and IL-6 are correlated with insulin resistance, impaired glucose tolerance, and type 2 DM [52, 53]. Both cytokines have been reported to reduce insulin signaling via suppressing the phosphorylation of protein kinase B (PKB/AKT) and insulin receptor substrate- (IRS-) 1 [54–56]. Therefore, the antihyperglycemic and insulin sensitizing effects of *M. quadrangula* are mediated, at least in part, via its antioxidant and anti-inflammatory activities. The ability of *M. quadrangula* extract to reduce inflammation has been recently reported in our work where it decreased the serum levels of proinflammatory cytokines in HCD-fed rats [23].

We assumed that the upregulation of adiponectin mediates the antidiabetic effect of *M. quadrangula* extract in HFD/STZ diabetic rats. Thus, we measured the effect of *M. quadrangula* extract in serum adiponectin and the adiponectin expression in the liver of HFD/STZ diabetic rats. In the present study, diabetic rats showed a significant decrease in serum adiponectin and hepatic adiponectin gene expression as previously reported [37, 39]. Lowered serum levels of adiponectin were found to be associated with insulin resistance and the etiology of obesity and type 2 DM [57]. Diabetic rats treated with *M. quadrangula* extract for 4 weeks showed increased levels of serum adiponectin and its hepatic expression. These results were correlated with an improved glucose tolerance, insulin sensitivity, hepatic glucose output, and peripheral glucose uptake. Adiponectin has been reported to stimulate AMP-activated protein kinase resulting in an increased insulin sensitivity and regulation of glucose metabolism [58]. In addition, adiponectin decreases the expression of glucose-6-phosphatase and phosphoenolpyruvate carboxylase, leading to a decreased hepatic glucose output via inhibition of hepatic gluconeogenesis [58]. Furthermore, adiponectin can activate peroxisome proliferator activated receptor- (PPAR-) *α*, decrease the hepatic and skeletal muscle triglyceride content [59], and enhance the oxidation of muscle fat through inhibition of acetyl-CoA carboxylase inhibition [60].

In conclusion, our results showed for the first time that *M. quadrangula* extract improves insulin sensitivity and glucose tolerance in HFD/STZ type 2 diabetic rats. *M. quadrangula* increased peripheral glucose uptake, improved lipid profile, suppressed hepatic glucose output, and prevented oxidative stress and inflammation in diabetic rats. In addition, *M. quadrangula* extract increased the serum adiponectin levels and adiponectin gene expression in the liver of the diabetic rats. These findings point to the role of adiponectin in mediating the antidiabetic effect of *M. quadrangula*; however, further studies to determine its exact mechanism of action are recommended.

## Figures and Tables

**Table 1:** Primers used for qPCR.

	Forward primer	Reverse primer
Adiponectin	5′-CCACCCAAGGAAACTTGTGC-3′	5′-CCCGGTATCCCATTGTGACC-3′
GAPDH	5′-AACTTTGGCATCGTGGAAGG-3′	5′-CCCGGTATCCCATTGTGACC-3′

## Data Availability

The data used to support the findings of this study are available from the corresponding author upon request.
